# World Trade Center Site Exposure Duration Is Associated with Hippocampal and Cerebral White Matter Neuroinflammation

**DOI:** 10.1007/s12035-022-03059-z

**Published:** 2022-10-15

**Authors:** Chuan Huang, Minos Kritikos, Mario Serrano Sosa, Thomas Hagan, Alan Domkan, Jaymie Meliker, Alison C. Pellecchia, Stephanie Santiago-Michels, Melissa A. Carr, Roman Kotov, Megan Horton, Sam Gandy, Mary Sano, Evelyn J. Bromet, Roberto G. Lucchini, Sean A. P. Clouston, Benjamin J. Luft

**Affiliations:** 1 Department of Radiology, Renaissance School of Medicine at Stony Brook, Stony Brook, NY, USA; 2 Department of Psychiatry, Renaissance School of Medicine at Stony, Brook University, Stony Brook, NY, USA; 3 Department of Biomedical Engineering, Stony Brook University, Stony Brook, NY, USA; 4 Program in Public Health and Department of Family, Population, and Preventive Medicine, Renaissance School of Medicine at Stony Brook University, Health Sciences Center, 101 Nichols Rd#3-071, Stony Brook, NY 11794, USA; 5 Department of Physiology and Biophysics, Stony Brook University, Stony Brook, NY, USA; 6 Stony Brook World Trade Center Wellness Program, Renaissance School of Medicine at Stony, Brook University, Stony Brook, NY, USA; 7 Department of Environmental Medicine and Public Health, Icahn School of Medicine at Mount Sinair, New York, NY, USA; 8 Center for Cognitive Health and Department of Neurology, Icahn School of Medicine at Mount Sinai, New York, NY, USA; 9 Department of Psychiatry and Mount Sinai Alzheimer’s Disease Research Center, Icahn School of Medicine at Mount Sinai, New York, NY, USA; 10 James J Peters VA Medical Center, 130 West Kingsbridge Road, Bronx, NY 10468, USA; 11 Department of Medicine, Renaissance School of Medicine at Stony Brook University, Stony Brook, NY, USA

**Keywords:** World Trade Center, Exposure, Neuroinflammation, Free water fraction, Diffusion tensor imaging, Cognitive impairment, Early-onset dementia

## Abstract

Responders to the World Trade Center (WTC) attacks on 9/11/2001 inhaled toxic dust and experienced severe trauma for a prolonged period. Studies report that WTC site exposure duration is associated with peripheral inflammation and risk for developing early-onset dementia (EOD). Free Water Fraction (FWF) can serve as a biomarker for neuroinflammation by measuring in vivo movement of free water across neurons. The present case-controlled study aimed to examine associations between WTC site exposure duration as well as EOD status with increased hippocampal and cerebral neuroinflammation. Ninety-nine WTC responders (mean age of 56) were recruited between 2017 and 2019 (*N* = 48 with EOD and 51 cognitively unimpaired). Participants were matched on age, sex, occupation, race, education, and post-traumatic stress disorder (PTSD) status. Participants underwent neuroimaging using diffusion tensor imaging protocols for FWF extraction. Region of interest (ROI) analysis and correlational tractography explored topographical distributions of FWF associations. Apolipoprotein-e4 allele (*APOE*ε4) status was available for most responders (*N* = 91). Hippocampal FWF was significantly associated with WTC site exposure duration (*r* = 0.30, *p* = 0.003), as was cerebral white matter FWF (*r* = 0.20, *p* = 0.044). ROI analysis and correlational tractography identified regions within the limbic, frontal, and temporal lobes. Hippocampal FWF and its association with WTC exposure duration were highest when the *APOE*ε4 allele was present (*r* = 0.48, *p* = 0.039). Our findings demonstrate that prolonged WTC site exposure is associated with increased hippocampal and cerebral white matter neuroinflammation in WTC responders, possibly exacerbated by possession of the *APOE*ε4 allele.

## Introduction

Alzheimer’s disease (AD) and other related dementias (ADRD) are among the most common causes of death in old age [1], with cognitive impairment (CI) as the most common early symptom [2, 3]. ADRD arises from neuropathology [4], including neuroinflammation [5], and is characterized by neurodegeneration in the frontal, parietal, and temporal lobes [6, 7], with the hippocampus as a centrally affected region [8]. Hippocampal degeneration in ADRD is often accompanied with cognitive decline such as hallmark memory disruptions [9, 10]. Other etiologies for ADRD often include risk factors such as older age (> 65 years), family history of dementia and/or *APOE*ε4 allele possession, and cardiovascular disease [1]. However, early-onset dementias (EOD) can be sporadic and can manifest with late-life dementia symptoms during midlife (40–65 years on average), potentially leading to more severe outcomes later [11]. Toxic exposures, such as repeated inhalations of nPM < 2.5 μm nano-particulate matter (nPM) pollutants, can also contribute to accelerated cognitive decline [12, 13], and are associated with neuroinflammatory and neurodegenerative processes [14–16]. Furthermore, *APOE*ε4 allele possession can compromise blood–brain barrier (BBB) permeability and vulnerability to nPM infiltration from repeated exposures, leading to worse outcomes [13, 17].

During the attacks on the World Trade Center (WTC) on 9/11/2001, WTC responders, now at midlife, were involved in the search, rescue, and clean-up efforts and endured a multitude of exposures, such as repeated inhalations to toxic aerosolized nPM, such as smoke, dioxins, and polycyclic aromatic hydrocarbons [13, 18, 19]. Two decades since 9/1t1, approximately 14% of WTC responders are experiencing early-onset mild cognitive impairment (MCI) or EOD [20, 21], which is associated with prolonged WTC site exposure duration [18], and carrying the *APOE*ε4 allele [20, 22]. More specifically, we identified that WTC responders with EOD had reduced cerebellar [23] and cortical thickness in the frontal, parietal, and temporal lobes [24], while those with MCI had altered white matter integrity [25] and reduced hippocampal volume [26]. Additionally, chronic post-traumatic stress disorder (PTSD) had reduced cortical complexity [27], systemic inflammation [28] with monocytic signaling [29], and positron emission tomography (PET) translocator protein (TSPO) radioligand positive scans that confirmed the presence of activated glia throughout the brain [30]. Taken together, we interpret these findings at large as an inevitable consequence of their novel exposures at the WTC site, insofar that a substantial portion of WTC responders are now experiencing an emerging clinical condition based on an undetermined, but early-onset ADRD type of neurodegenerative process, with a neuroinflammatory basis [20, 23].

Free water fraction (FWF) is an MRI diffusion tensor imaging (DTI) technique that has been previously shown to estimate underlying neuroinflammation in vivo and its association with CI [31–33]. FWF can be derived from diffusion MRI data assuming a two-compartment model—extracellular water and tissue fraction [34–37]. A known byproduct of neuroinflammation is when the brain clears interstitial extraneuronal spaces resulting in localized changes to diffusing free water, which is what FWF measures [38–42]. Thus, there is increasing evidence demonstrating that FWF can be used as a marker for neuroinflammation [35, 41], and that it is often associated with CI [31–33]. Moreover, measuring FWF in the hippocampus can potentially serve as a sensitive biomarker of neuroinflammation for detecting ADRD, even in the absence of volumetric changes [43]. FWF is a superior measure for neuroinflammation, as other diffusion metrics such as fractional anisotropy or mean diffusivity are known to be sensitive to both neuroinflammation and other confounding pathology [38].

Considering that our prior work suggests associations between EOD, neuroinflammation, and WTC site exposure duration in this population, the primary aim of this study was to examine whether FWF in the hippocampus, cerebral white, or cerebral gray matter was associated with WTC site exposure duration. Our secondary aim was to examine whether responders with EOD displayed higher FWF in these regions when compared to cognitively unimpaired (CU) controls. Our third aim was to explore a region of interest (ROI) based topographic distribution of FWF levels to highlight which neuroanatomical regions display higher neuroinflammation.

## Methods

### Population

The WTC Health Program (WTCHP) was established in 2002 and is in charge of monitoring over 50,000 responders [44] via a protocol described in depth elsewhere [45]. As previously described [46], all WTC responders are eligible to attend annual health monitoring visits and receive treatment for WTC-certified conditions [47]. Eligible WTC responders continue to participate in an ongoing epidemiologic study of cognitive aging [48]. In the present report, WTC responders were contacted if consent had been previously collected at enrollment to permit for possible recruitment in future studies and if their patient characteristics matched necessary inclusion/exclusion criteria.

We employed a two-by-two study design with four groups including EOD (present/absent) and PTSD (present/absent). Inclusion criteria at the time of screening were ages 44 to 65, fluency in English, current cognitive assessment indicative of cognitive unimpaired (CU), EOD, or PTSD. While not an inclusion/exclusion criterion, all but one responder with CI had functional limitations consistent with mild dementia. Responders whose case status was not confirmed at during re-assessment were excluded from further participation in this study. To control for certain characteristic differences across groups, responder participants were matched on age, sex, race/ethnicity, occupation, and education at the time of screening.

Exclusion criteria included: history of psychosis, history of diagnosed neurological conditions, including major stroke, multiple sclerosis, and Parkinson’s disease; head injury during their WTC response efforts, history of military head trauma including combustive blasts; current liver disease; and current use of cognitively active medications. Subjects also had to satisfy eligibility criteria for MRI scanning including body mass index ≤ 40, no known claustrophobia, and no known metal implants or shrapnel that were not deemed MRI-safe.

In total, 598 WTC responders on file were initially contacted upon matching initial inclusion criteria with 176 of which were scheduled for screening visits after expressing participation interest. Approximately 88% of scheduled responders (*n* = 156) completed screening visits, wherein 27.6% (*n* = 43) were ineligible to continue and excluded, with 7.7% (*n* = 13) declining further participation. Finally, 100 WTC responders participated in neuroimaging; with only 99 responders completing the scan protocol. The time between screening and the neuroimaging scan was 26 days on average (standard deviation [SD] = 17.2; interquartile range = 14–35 days).

### Early-Onset Dementia

Dementia status was diagnosed algorithmically following guidelines by the National Institute on Aging and Alzheimer’s Association [49]. EOD was operationalized using the Montreal Cognitive Assessment (MoCA ≤ 20) [50], a widely used and validated measure of cognitive status developed to identify age-related CI objectively and reliably. CU status was operationalized when no evidence of CI (MoCA ≥ 26) was present.

### Matching Criteria

PTSD status was operationalized using the Structured Clinical Interview for the DSM-IV [SCID-IV] [51], a semi-structured interview, administered by trained clinical interviewers. Severity for PTSD symptoms such as re-experiencing, avoidance, hyperarousal, and numbing was measured by examining subscale scores in a WTC specific version of the PTSD checklist (PCL17-S). Other matching criteria included age, sex, race/ethnicity (White, Black, Hispanic, Other), occupation (law enforcement versus other), and educational attainment (high school or less, some college, or university degree).

### Image Acquisition

Three-dimensional T1-weighted magnetization-prepared rapid gradient echo (MPRAGE) images were acquired on a 3 T Siemens Biograph mMR TR/TE/TI = 1900/2.4/900 ms; flip angle = 9°; acquisition matrix = 256 × 256; voxel resolution = 1 × 1 × 1 mm at Icahn School of Medicine, Mount Sinai, NY. For incidental neuropathology screening, T2-weighted anatomical scans used a turbo spin-echo pulse sequence (34 axial slices), TR/TE = 6170/96 ms; flip angle = 150°; acquisition matrix = 320 × 320; voxel size = 0.36 × 0.36 × 3 mm and were read by a board-certified radiologist to determine any incidental neurological findings—no incidental findings were identified. DTI sequences were performed with the following parameters: TE/TR = 87.6/4680 ms, *b* value = 1000, 64 diffusion directions, in-plane resolution = 2 × 2 mm^2^, slice thickness = 2 mm, matrix size = 128 × 128, multiband factor = 2.

### Image Processing

Following acquisition, DTI images were visually checked for major image or significant motion artifacts, with all 99 images passing inspection. Diffusion data from the DTI sequences were post-processed for each subject and involved motion and eddy correction [52], followed by generation of an FWF map. While the DiPy toolbox [53] has previously been utilized for multi-shell FWF generation [54–56], single-shell FWF images were generated using the recent extension to DiPy proposed by Golub et al. [37]. Our team utilized default initialization for FWF calculation with 200 iterations and a learning rate of 0.0005 as has been done previously [37] with the spatial regularization operator turned off at half iterations. Independent isotropic values (ISO), which measures the density of isotropically diffusing water calculated from q-sampling and is associated with FWF at lower *b*-values and represents the density of free water diffusion metrics *along* the subsection of a fiber pathway [37, 57–60], were calculated to examine subregional results using correlational tractography [61].

### Correlational Tractography

Correlational tractography analysis was conducted to measure how ISO in white matter tracts correlated with WTC site exposure duration. Q-space diffeomorphic reconstruction was implemented in DSI-Studio to reconstruct subject data into MNI space and to then calculate a spin distribution function [62]. Nonparametric Spearman’s partial correlations were derived and a t-score threshold of 3 was operationalized as our threshold. Deterministic fiber tracking algorithms were utilized to obtain correlational tractography and whole brain seeding. Tracks were filtered by topology-informed pruning with 4 iterations using a length threshold of 20 voxels.

### Region of Interest (ROI) Parcellation

**Fig. 1:**
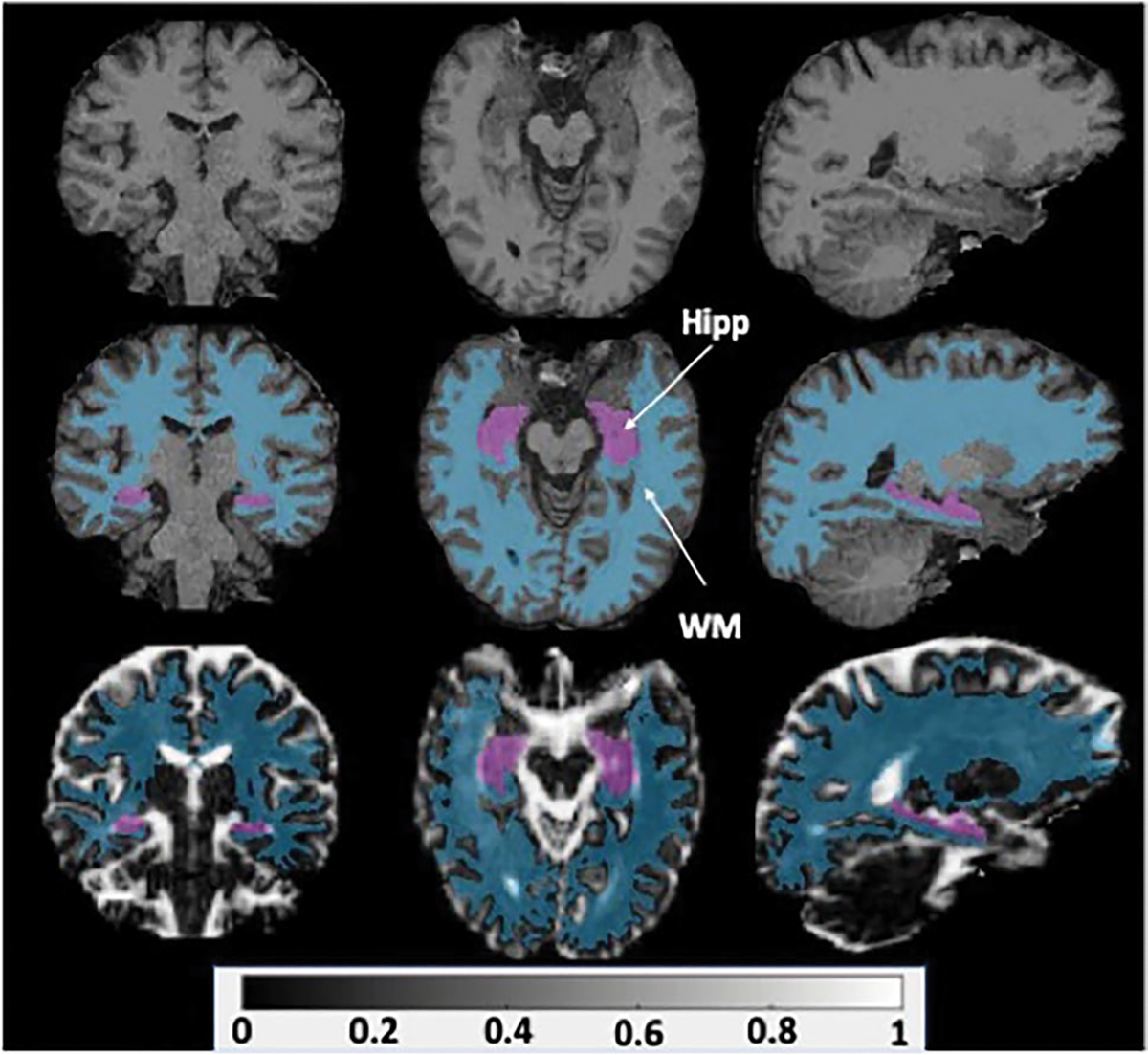
T1 volumes (top) were used as references for linear and affine FWF data registration. A sample registered FWF volume (bottom) is shown with hippocampal (Hipp) and white matter (WM) overlays calculated from FreeSurfer regional parcellation (middle)

Following linear and affine registration of each scan to a corresponding structural T1 reference image, ROI parcellation was conducted in FreeSurfer 7 using the Desikan Killiany (DK) atlas. A sample of the resulting hippocampal and white matter overlays from the registration pipeline can be seen in Fig. 1. The final output included 68 ROIs with FWF volumes that were estimated using FMRIB Software Library FLIRT tool [63].

### WTC Exposure Duration

Upon enrollment into the larger WTC epidemiologic study, all responders undergo a baseline interviewer-administered exposure assessment questionnaire to record exposures to harmful physical and psychological conditions during their WTC recovery efforts [45]. In the present study, we examined the variable of time (hours) spent at the WTC recovery site [47, 64].

### APOEε4 Status

To determine APOEε4 status, DNA was extracted from blood collected during visits during study baseline. Single nucleotide polymorphism genotyping was performed using the Agena iPLEX kit with processing completed at Roswell Park Laboratories. Among the 99 responders with imaging data available, the APOEε4 allele was genotyped in 92% of responders (*n* = 91, 70 APOEε4−, 21 APOEε4 +). APOEε4 + was operationalized when the responder possessed either the homozygous or heterozygous allele.

### Statistical Analyses

Descriptive characteristics were provided using mean and standard deviations, or frequencies and percentages were noted. Welch’s *t*-tests and *χ*^2^ tests examined differences in matching and diagnostic variables across study groups. A multi-factor analysis of variance (ANOVA) was used to study the association between EOD status, and WTC site exposure duration. Pearson’s correlation was used to study the association between WTC site exposure duration and FWF. Sensitivity analyses examined Spearman’s correlations between FWF and PTSD status, and PTSD symptom severity. Statistical significance was set at *α* = 0.05, with analyses reporting nominal and false discovery rate (FDR) adjusted *p*-values were noted [65].

## Results

### Sample Characteristics

As per our recruitment design, groups stratified by cognitive status did not differ on matching criteria, such as age, sex, race, PTSD status, education, or occupation (see Table 1) thereby negating the need to further control for these variables as potential confounders. Nevertheless, the results with age/sex/education correction are also provided.

### FWF and EOD Status

EOD status was not significantly associated with hippocampal mean FWF (*p* = 0.656, Cohen’s *d* = − 0.09), nor cerebral gray matter FWF (*p* = 0.668, Cohen’s *d* = 0.09), with cerebral white matter FWF approaching significance (*p* = 0.060, Cohen’s *d* = − 0.38). The results stayed the same after age/sex/education corrections; the corresponding *p*-values were 0.314, 0.088, and 0.833.

### FWF and WTC Site Exposure Duration

**Fig. 2:**
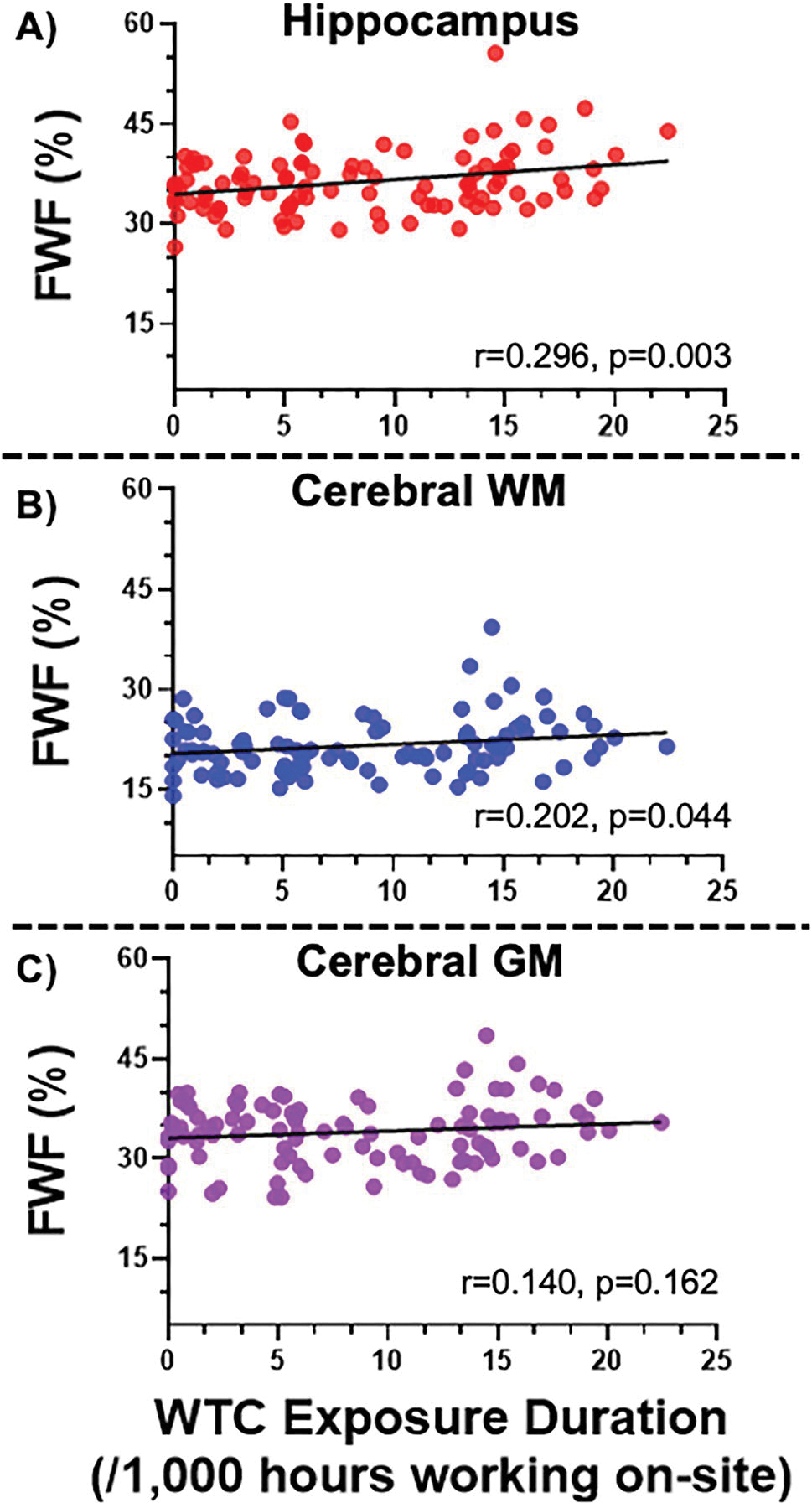
FWF and WTC site exposure duration. Scatter plot showing Pearson’s correlation coefficients from Table 2 between World Trade Center site exposure duration (hours) and free water fraction (FWF) in **A** hippocampus (*r* = 0.30, *p* = 0.003), **B** cerebral white matter (*r* = 0.20, *p* = 0.044), and **C** cerebral gray matter (*r* = 0.14, *p* = 0.162). The corresponding result after age/sex/education corrections is **A** *r* = 0.33, *p* = 0.0009, **B** *r* = 0.21, *p* = 0.033, **C** *r* = 0.17, *p* = 0.092

Statistically significant correlations were identified between WTC site exposure duration and hippocampal and cerebral white matter FWF, but not cerebral gray matter (see Fig. 2).

### Cerebral White Matter ROI FWF and WTC Site Exposure Duration

**Fig. 3:**
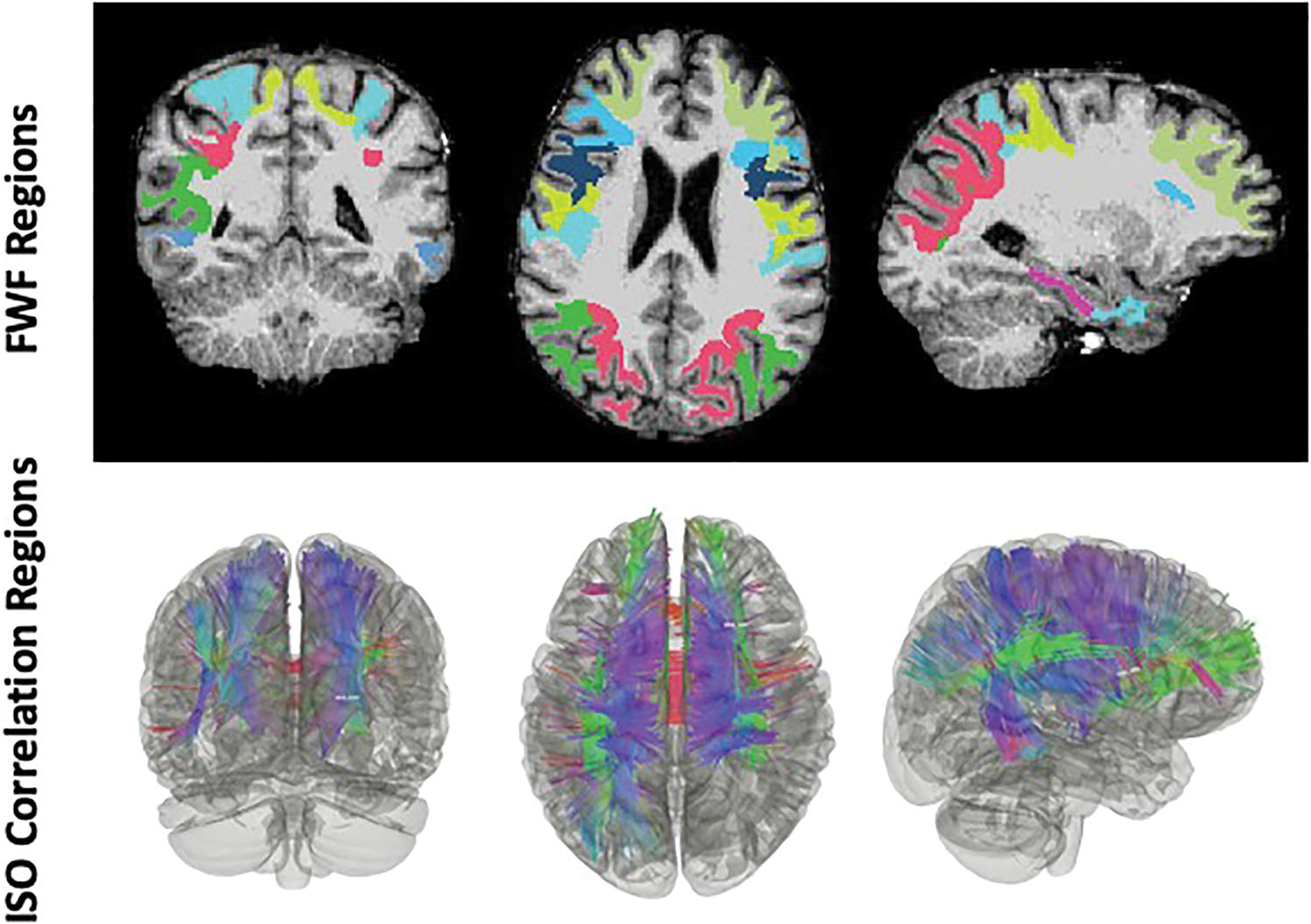
Correlational tractography. Free water fractions (FWF) within regions of interest (ROIs) from Table 2 (uncorrected) and ISO from correlational tractography analysis (bottom panel, t-threshold = 3, FDR = 0.011), were both significantly correlated with WTC site exposure duration and overlap in many ROIs, such as frontal and parietal regions

Cerebral white matter was parcellated into unilateral regions from the DK atlas and the mean FWF of several ROIs, including the parahippocampus (*r* = 0.28, *p* = 0.005), were found to be significantly correlated with WTC site exposure duration (hours) (see Table 2 and Fig. 3).

### Correlational Tractography

Correlational tractography identified a positive association between ISO diffusion and WTC site exposure duration (hours) (T-threshold = 3, FDR = 0.011, with age/sex/education corrected), in ROIs that overlap with ROIs that were significant in Table 2 (see Fig. 3).

### APOEε4 status, FWF, and WTC Site Exposure Duration

Spearman’s correlations between WTC site exposure duration (hours) and hippocampal and cerebral white and gray matter FWF were performed for the two subgroups with differing APOEε4 status, regardless of cognitive status. Cerebral white and gray matter FWF was not significantly associated with WTC site exposure duration (hours) (data not shown), but hippocampal FWF was, and the correlation coefficients were higher in the APOEε4 + group (*r* = 0.48, *p* = 0.039, *N* = 21) when compared to the APOEε4- group (*r* = 0.25, *p* = 0.039, *N* = 70).

### FWF and PTSD Status

No significant associations between hippocampal and cerebral white and gray matter FWF were found when examining PTSD status (data not shown).

## Discussion

As has been previously demonstrated by our team, the WTC population is at high risk of developing cognitive impairment through a neurodegenerative process [20]. We previously suggested that neuroinflammatory mechanisms might help to explain the association between WTC exposures and the risk of MCI in this cohort. The present study primarily aimed to test whether WTC site exposure duration, EOD status, and PTSD status, were associated with higher levels of the neuroinflammatory neuroimaging marker FWF, specifically in the hippocampus as well as the cerebral white and gray matter of WTC responders. In summary, we did not identify a significant association of FWF with hippocampal and cerebral white and gray matter FWF with EOD, nor PTSD status. However, we report here, for the first time, a significant correlation between hippocampal and cerebral white matter FWF with WTC site exposure duration that may be exacerbated by possession of the APOEε4 allele. Exploratory ROI-based analysis of cerebral white matter FWF further identified that parahippocampal and entorhinal white matter FWF had the highest correlation coefficients with WTC site exposure duration, followed by the transverse temporal which together with the prior two ROIs remained significant after age/sex/education adjustment, and the pars triangularis regions.

We did not identify a correlation between FWF in the hippocampus and WTC EOD status, which is in line with prior findings of how WTC site exposure was *not* correlated with MCI after controlling for APOEε4 status [47]. Hippocampal disruptions are a hallmark for patients with AD and certain related dementias [66], and our findings here suggest that WTC responders with EOD are not as significantly affected by hippocampal disruptions as these patients. Further support is suggested from the association between cerebral white matter mean FWF and EOD, which approached significance. Taken together with the association of cerebral FWF with WTC site duration, a larger sample size may reveal that cerebral white matter FWF is also associated with WTC responder EOD status.

Extensive literature has detailed the sensitivity and involvement of hippocampal changes to cognitive decline [9, 26], both through aging and disease progression. In this work, even though hippocampal FWF was not associated with EOD status, a statistically significant positive correlation was found between hippocampal FWF and WTC site exposure duration. Furthermore, this finding is in line with the higher hippocampal glial activation we previously found in WTC responders with MCI [67]. Taken together, this suggests that responders with higher WTC site exposure duration may display more severe hippocampal neuroinflammation, possibly due to prolonged exposures to nPM inhalations at the WTC disaster site [13, 19]. For example, among the nPM present at Ground Zero, thallium—a toxic heavy metal capable of targeting and damaging the nervous system—was existent in concentrations 1,000 times higher than evident in unexposed parts of New York City [68, 69].

Cerebral white matter FWF and WTC site exposure duration were also found to significantly correlated, with several ROIs involved, mostly within the temporal and limbic areas such as the transverse temporal, entorhinal, and parahippocampal formation. Moreover, clusters of correlations with WTC exposure duration were also found in ROIs within the parietal and frontal lobes. The highest correlation was in the pars triangularis, which remained significant after age/sex/education adjustment, followed by the pars orbitalis and opercularis, the precentral, and then the postcentral, inferior, and superior parietal. These ROIs are in line with cortical thinning previously identified in WTC responders with EOD [24], and our work here now suggests that neuroinflammation associated with prolonged WTC site exposure duration may have been a mechanism for neurodegeneration. Taken together, associations of WTC exposure duration with FWF in key cerebral ROIs suggest that prolonged durations at the site may have led to widespread neuroinflammatory activation and downstream outcomes. This finding is also in line with prior literature suggesting toxic effects of nPM on the BBB [70, 71], as well as neuroinflammatory consequences of nPM inhalations [15], which may be placing affected WTC responders with higher WTC exposure duration at increased risk for developing neuropathological phenomena [13].

APOEε4 is known to increase the relative risk for CI and dementia, and we identified that APOEε4 status was associated with hippocampal FWF and WTC site exposure duration, with nearly twice the effect size in APOEε4 + responders (APOEε4 + : *r* = 0.48 versus APOEε4-: *r* = 0.25). This suggests that the presence of the APOEε4 allele may moderate the correlation between hippocampal FWF and WTC site exposure and is consistent with prior work showing that incidence of MCI was higher among APOEε4 + WTC responders with prolonged WTC exposures [47]. This is in line with studies demonstrating that *APOE*ε4 allele possession can compromise blood–brain barrier (BBB) permeability and vulnerability to nPM infiltration from repeated exposures, leading to worse outcomes [13, 17].

WTC responders are known to suffer from chronic PTSD [72], which is also a risk factor for cognitive dysfunction [20]. Approximately half of our study responder participants herein had clinically significant levels of PTSD and related symptoms. However, we found no associations between hippocampal and cerebral white and gray matter FWF and PTSD, suggesting that the effects of PTSD may engage different risk mechanisms for CI in those affected. Indeed, several other studies with WTC responders have similarly shown that PTSD was not associated with degree or type of neurodegeneration after accounting for CI status [23, 24, 26]. Therefore, our present findings suggest that FWF could be an in vivo neuroinflammatory marker for WTC exposure site duration independent of PTSD status, and possibly moderated by APOEε4 status.

### Limitations

In this work, we did not have APOEε4 status for all responders and the distribution of APOEε4 ± responders was imbalanced (*n* = 21 versus *n* = 70). However, we observed that APOEε4 status may moderate the association between hippocampal neuroinflammation and WTC site exposure. Nevertheless, a future prospective investigation controlling for APOEε4 status is needed to validate this finding. Another limitation of this study are the mathematical limitations of FWF model for single-shell diffusion data as prior diffusion studies have suggested to generate FWF images using multi-shell data for the fitting of the two-compartmental bi-tensor model instead [35, 73]. However, other studies have demonstrated reliability across both single-shell and multi-shell algorithms [37, 74], but care is needed to interpret the data [37]. Finally, the present study did not test for network-based structural correlations with WTC site exposure duration, as outlined in this study [75], and although we have performed some diffusion-based studies in prior publications [27, 76], future neuroimaging studies of WTC responders should consider these approaches.

## Conclusions

This study identified that hippocampal and cerebral white matter neuroinflammation, as measured by FWF derived from DTI-MRI, is associated with WTC site exposure duration for a sample of 99 WTC responders with and without EOD, regardless of PTSD status. Several key white matter ROIs were involved, suggesting a widespread neuroinflammatory process that may mechanistically underlie our prior findings of gray matter neurodegeneration in those same regions. WTC site exposure-related prolonged inhalations of nPM may be responsible for this neuroinflammation that seems to be independent of PTSD and may be exacerbated by possessing the APOEε4 allele. Further investigation is needed to validate and elaborate on the findings reported herein.

## Figures and Tables

**Table 1:** World Trade Center (WTC) responder sample characteristics

Characteristic	Overall	Cognitively unimpaired	Early-onset dementia	*t*-test/*x^2^*
	*N* = 99	*N* = 51	*N* = 48	*p*

Age	56.38 (0.52)	56.42 (0.63)	56.32 (0.86)	0.926
PTSD—DSM-IV (SCID-IV)				
No	52.50%	53.90%	51.10%	0.782
Yes	47.50%	46.20%	48.90%	
APOEε4 status (*n* = 91)				
APOEε4 +	21.21%	21.57%	20.83%	0.227
APOEε4−	67.68%	72.55%	62.50%	
Unknown	11.11%	5.88%	16.67%	
Sex				
Male	85.30%	80.80%	76.60%	0.612
Female	14.70%	19.20%	23.40%	
Minority status				
White	77.80%	86.50%	68.10%	0.087
Black	10.10%	5.80%	14.90%	
Hispanic	12.10%	7.70%	17.00%	
Occupation				
NYPD	56.60%	60.80%	52.10%	0.383
Other	43.40%	39.20%	47.90%	
Education				
High school or less	23.20%	17.70%	29.20%	0.359
Some college	47.50%	49.00%	45.80%	
University degree	29.30%	33.30%	25.00%	

Values expressed as means (standard deviations) or percentages (%); *p*-values from Welch’s *t*-tests or chi-squared test comparisons examine the extent to which noted characteristics differ across groups; *PTSD,* post-traumatic stress disorder; *APOEε4,* the ε4 allele of the Apolipoprotein E (APOE) gene; *DSM-IV (SCID-IV),* Structured Clinical Interview using the Diagnostic and Statistical Manual IV; *NYPD,* New York Police Department

**Table 2:** Cerebral White Matter ROI FWF and WTC site exposure duration

*Lobe*	Regions of Interest	r	p	r#	P#

***Frontal***	Caudal Middle Frontal	0.10	0.302	0.09	0.430
	Frontal Pole	−0.03	0.746	−0.03	0.774
	Lateral Orbitofrontal	0.15	0.152	0.15	0.122
	Medial Orbitofrontal	0.16	0.109	0.13	0.177
	Pars Opercularis	**0.22**	**0.028**	0.17	0.082
	Pars Orbitalis	0.21	**0.040**	0.17	0.088
	Pars Triangularis	**0.27**	**0.008** *	**0.23**	**0.021**
	Rostral Middle Frontal	**0.21**	**0.038**	0.19	0.057
	Superior Frontal	0.15	0.139	0.12	0.232
	Precentral	**0.23**	**0.026**	0.15	0.135

***Limbic***	Caudal Anterior Cingulate	**0.19**	0.059	0.19	0.053
	Rostral Anterior Cingulate	0.15	0.153	0.11	0.288
	Isthmus Cingulate	0.10	0.320	0.11	0.284
	Insula	0.18	0.078	0.16	0.099
	Parahippocampal	**0.28**	**0.005** *	**0.32**	**<0.001**
	Posterior Cingulate	0.13	0.217	0.10	0.290

***Temporal***	Banks Superior Temporal Sulcus	0.16	0.116	0.14	0.156
	Entorhinal	**0.28**	**0.006** *	**0.20**	**0.045**
	Inferior Temporal	0.19	0.060	0.12	0.209
	Middle Temporal	0.21	**0.043**	**0.18**	**0.063**
	Superior Temporal	0.18	0.071	0.18	0.062
	Temporal Pole	0.11	0.279	0.05	0.641
	Transverse Temporal	**0.27**	**0.007** *	**0.24**	**0.014**

***Parietal***	Inferior Parietal	**0.24**	**0.020**	0.17	0.083
	Paracentral	0.14	0.181	0.09	0.343
	Postcentral	**0.20**	**0.045**	0.10	0.297
	Precuneus	0.18	0.079	0.13	0.198
	Superior Parietal	**0.22**	**0.032**	0.15	0.124
	Supramarginal	0.19	0.070	0.13	0.196
	Pericalcarine	0.06	0.533	0.02	0.826
	Fusiform	0.15	0.142	0.14	0.156
	Cuneus	0.14	0.161	0.08	0.430
	Lateral Occipital	0.08	0.435	0.04	0.725
	Lingual	0.13	0.223	0.11	0.284

Correlations between World Trade Center site exposure duration (hours) and cerebral white matter free water fraction in 68 regions of interest (ROIs) from the Desikan-Killiany Atlas. Significant correlations are highlighted in **Bold**

the * denotes ROIs that remained statistically significant following false discovery rate correction (FDR = 0.10)

the # denotes age/sex/education corrected

## Data Availability

Medical information for these participants is protected, so only processed de-identified data will be made available upon receipt of reasonable request to the corresponding author.

## References

[1] Alzheimer’s Association (2021) 2021 Alzheimer’s disease facts and figures. Alzheimers Dement 17(3):327–406. 10.1002/alz.12328 33756057

[2] Backman L, Small BJ, Fratiglioni L (2001) Stability of the preclinical episodic memory deficit in Alzheimer’s disease. Brain 124(Pt 1):96–102. 10.1093/brain/124.1.96 11133790

[3] Rodrigue KM, Kennedy KM, Devous MD Sr, Rieck JR, Hebrank AC, Diaz-Arrastia R, Mathews D, Park DC (2012) beta-Amyloid burden in healthy aging: regional distribution and cognitive consequences. Neurology 78(6):387–395. 10.1212/WNL.0b013e318245d295 22302550 PMC3280058

[4] Jack CR Jr, Bennett DA, Blennow K, Carrillo MC, Dunn B, Haeberlein SB, Holtzman DM, Jagust W (2018) NIA-AA Research Framework: Toward a biological definition of Alzheimer’s disease. Alzheimers Dement 14(4):535–562. 10.1016/j.jalz.2018.02.018 29653606 PMC5958625

[5] Parbo P, Ismail R, Hansen KV, Amidi A, Marup FH, Gottrup H, Braendgaard H, Eriksson BO (2017) Brain inflammation accompanies amyloid in the majority of mild cognitive impairment cases due to Alzheimer’s disease. Brain 140(7):2002–2011. 10.1093/brain/awx120 28575151

[6] Bakkour A, Morris JC, Dickerson BC (2009) The cortical signature of prodromal AD: regional thinning predicts mild AD dementia. Neurology 72(12):1048–1055. 10.1212/01.wnl.0000340981.97664.2f 19109536 PMC2677470

[7] Im K, Lee JM, Seo SW, Yoon U, Kim ST, Kim YH, Kim SI, Na DL (2008) Variations in cortical thickness with dementia severity in Alzheimer’s disease. Neurosci Lett 436(2):227–231. 10.1016/j.neulet.2008.03.032 18400396

[8] Burton EJ, Barber R, Mukaetova-Ladinska EB, Robson J, Perry RH, Jaros E, Kalaria RN, O’Brien JT (2008) Medial temporal lobe atrophy on MRI differentiates Alzheimer’s disease from dementia with Lewy bodies and vascular cognitive impairment: a prospective study with pathological verification of diagnosis. Brain 132(1):195–203. 10.1093/brain/awn298 19022858

[9] Bettio LE, Rajendran L, Gil-Mohapel J (2017) The effects of aging in the hippocampus and cognitive decline. Neurosci Biobehav Rev 79:66–86 28476525 10.1016/j.neubiorev.2017.04.030

[10] Morris RG, Garrud P, Rawlins JN, O’Keefe J (1982) Place navigation impaired in rats with hippocampal lesions. Nature 297(5868):681–683. 10.1038/297681a0 7088155

[11] Mendez MF (2019) Early-onset Alzheimer Disease and Its Variants. Continuum (Minneap Minn) 25(1):34–51. 10.1212/CON.0000000000000687 30707186 PMC6538053

[12] Kilian J, Kitazawa M (2018) The emerging risk of exposure to air pollution on cognitive decline and Alzheimer’s disease - Evidence from epidemiological and animal studies. Biomed J 41(3):141–162. 10.1016/j.bj.2018.06.001 30080655 PMC6138768

[13] JR, Luft BJ, Clouston SAP(2020) Acute versus Chronic Exposures to Inhaled Particulate Matter and Neurocognitive Dysfunction: Pathways to Alzheimer’s Disease or a Related Dementia. J Alzheimers Dis 78(3):871–886. 10.3233/JAD-200679 33074229 PMC7704925

[14] Cacciottolo M, Morgan TE, Saffari AA, Shirmohammadi F, Forman HJ, Sioutas C, Finch CE (2020) Traffic-related air pollutants (TRAP-PM) promote neuronal amyloidogenesis through oxidative damage to lipid rafts. Free Radical Biol Med 147:242–251 31883973 10.1016/j.freeradbiomed.2019.12.023 PMC7075030

[15] Babadjouni R, Patel A, Liu Q, Shkirkova K, Lamorie-Foote K, Connor M, Hodis DM, Cheng H (2018) Nanoparticulate matter exposure results in neuroinflammatory changes in the corpus callosum. PLoS One 13(11):e0206934 30395590 10.1371/journal.pone.0206934 PMC6218079

[16] Livingston G, Huntley J, Sommerlad A, Ames D, Ballard C, Banerjee S, Brayne C, Burns A (2020) Dementia prevention, intervention, and care: 2020 report of the Lancet Commission. Lancet 396(10248):413–446. 10.1016/S0140-6736(20)30367-6 32738937 PMC7392084

[17] Methia N, Andre P, Hafezi-Moghadam A, Economopoulos M, Thomas KL, Wagner DD (2001) ApoE deficiency compromises the blood brain barrier especially after injury. Mol Med 7(12):810–815 11844869 PMC1950012

[18] Clouston SAP, Hall CB, Kritikos M, Bennett DA, DeKosky S, Edwards J, Finch C, Kreisl WC (2021) Cognitive impairment and World Trade Centre-related exposures. Nat Rev Neurol. 10.1038/s41582-021-00576-8 PMC8938977 34795448

[19] Landrigan PJ, Lioy PJ, Thurston G, Berkowitz G, Chen LC, Chillrud SN, Gavett SH, Georgopoulos PG (2004) Health and environmental consequences of the world trade center disaster. Environ Health Perspect 112(6):731–739. 10.1289/ehp.6702 15121517 PMC1241968

[20] Clouston SAP, Hall CB, Kritikos M, Bennett DA, DeKosky S, Edwards J, Finch C, Kreisl WC (2022) Cognitive impairment and World Trade Centre-related exposures. Nat Rev Neurol 18(2):103–116. 10.1038/s41582-021-00576-8 34795448 PMC8938977

[21] Daniels RD, Clouston SAP, Hall CB, Anderson KR, Bennett DA, Bromet EJ, Calvert GM, Carreon T (2021) A Workshop on Cognitive Aging and Impairment in the 9/11-Exposed Population. Int J Environ Res Public Health 18(2):681. 10.3390/ijerph18020681 33466931 PMC7830144

[22] Clouston SA, Diminich ED, Kotov R, Pietrzak RH, Richards M, Spiro A III, Deri Y, Carr M (2019) Incidence of mild cognitive impairment in World Trade Center responders: long-term consequences of re-experiencing the events on 9/11/2001. Alzheimers Dement Diagn Assess Dis Monit 11(1):628–636 10.1016/j.dadm.2019.07.006 PMC6733774 31517025

[23] Clouston SAP, Kritikos M, Huang C, Kuan PF, Vaska P, Pellecchia AC, Santiago-Michels S, Carr MA (2022) Reduced cerebellar cortical thickness in World Trade Center responders with cognitive impairment. Transl Psychiatry 12(1):107. 10.1038/s41398-022-01873-6 35296637 PMC8927406

[24] Clouston S, Deri Y, Horton M, Tang C, Diminich ED, Pellecchia A, Carr M, Gandy S (2020) Reduced cortical thickness in World Trade Center responders with cognitive impairment: Neuroimaging/differential diagnosis. Alzheimers Dement 16:e039996 10.1002/dad2.12059 PMC7364857 32695871

[25] Huang C, Kritikos M, Clouston SAP, Deri Y, Serrano-Sosa M, Bangiyev L, Santiago-Michels S, Gandy S (2021) White Matter Connectivity in Incident Mild Cognitive Impairment: A Diffusion Spectrum Imaging Study of World Trade Center Responders at Midlife. J Alzheimers Dis 80(3):1209–1219. 10.3233/JAD-201237 33646156 PMC8150516

[26] Deri Y, Clouston SA, DeLorenzo C, Gardus JD III, Horton M, Tang C, Pellecchia AC, Santiago-Michels S (2021) Selective hippocampal subfield volume reductions in World Trade Center responders with cognitive impairment. Alzheimers Dement Diagn Assess Dis Monit 13(1):e12165 10.1002/dad2.12165 PMC8011041 33816755

[27] Kritikos M, Clouston SAP, Huang C, Pellecchia AC, Mejia-Santiago S, Carr MA, Kotov R, Lucchini RG (2021) Cortical complexity in world trade center responders with chronic posttraumatic stress disorder. Transl Psychiatry 11(1):597. 10.1038/s41398-021-01719-7 34815383 PMC8611009

[28] Rosen RL, Levy-Carrick N, Reibman J, Xu N, Shao Y, Liu M, Ferri L, Kazeros A (2017) Elevated C-reactive protein and posttraumatic stress pathology among survivors of the 9/11 World Trade Center attacks. J Psychiatr Res 89:14–21 28135632 10.1016/j.jpsychires.2017.01.007

[29] Kuan PF, Yang X, Clouston S, Ren X, Kotov R, Waszczuk M, Singh PK, Glenn ST (2019) Cell type-specific gene expression patterns associated with posttraumatic stress disorder in World Trade Center responders. Transl Psychiatry 9(1):1. 10.1038/s41398-018-0355-8 30664621 PMC6341096

[30] Deri Y, Clouston SAP, DeLorenzo C, Gardus JD 3rd, Bartlett EA, Santiago-Michels S, Bangiyev L, Kreisl WC (2021) Neuroinflammation in World Trade Center responders at midlife: A pilot study using [(18)F]-FEPPA PET imaging. Brain Behav Immun Health 16:100287. 10.1016/j.bbih.2021.100287 34589784 PMC8474562

[31] Dumont M, Roy M, Jodoin PM, Morency FC, Houde JC, Xie Z, Bauer C, Samad TA (2019) Free Water in White Matter Differentiates MCI and AD From Control Subjects. Front Aging Neurosci 11:270. 10.3389/fnagi.2019.00270 31632265 PMC6783505

[32] Pasternak O, Westin C-F, Bouix S, Seidman LJ, Goldstein JM, Woo T-UW, Petryshen TL, Mesholam-Gately RI (2012) Excessive extracellular volume reveals a neurodegenerative pattern in schizophrenia onset. J Neurosci Off J Soc Neurosci 32(48):17365–17372. 10.1523/JNEUROSCI.2904-12.2012 PMC3549332 23197727

[33] Pasternak O, Westin C-F, Dahlben B, Bouix S, Kubicki M (2015) The extent of diffusion MRI markers of neuroinflammation and white matter deterioration in chronic schizophrenia. Schizophr Res 161(1):113–118. 10.1016/j.schres.2014.07.031 25126717 PMC4277709

[34] Pierpaoli C, Jones D (2004) Removing CSF contamination in brain DT-MRIs by using a two-compartment tensor model. In: International Society for Magnetic Resonance in Medicine Meeting, 1215

[35] Pasternak O, Sochen N, Gur Y, Intrator N, Assaf Y (2009) Free water elimination and mapping from diffusion MRI. Magn Reson Med 62(3):717–730. 10.1002/mrm.22055 19623619

[36] Hoy AR, Koay CG, Kecskemeti SR, Alexander AL (2014) Optimization of a free water elimination two-compartment model for diffusion tensor imaging. Neuroimage 103:323–333. 10.1016/j.neuroimage.2014.09.053 25271843 PMC4312191

[37] Golub M, Neto Henriques R, Gouveia Nunes R (2021) Free-water DTI estimates from single b-value data might seem plausible but must be interpreted with care. Magn Reson Med 85(5):2537–2551. 10.1002/mrm.28599 33270935

[38] Pasternak O, Westin C-F, Bouix S, Seidman LJ, Goldstein JM, Woo T-UW, Petryshen TL, Mesholam-Gately RI (2012) Excessive extracellular volume reveals a neurodegenerative pattern in schizophrenia onset. J Neurosci 32(48):17365–17372 23197727 10.1523/JNEUROSCI.2904-12.2012 PMC3549332

[39] Schwartz M, Butovsky O, Brück W, Hanisch U-K (2006) Microglial phenotype: is the commitment reversible? Trends Neurosci 29(2):68–74 16406093 10.1016/j.tins.2005.12.005

[40] Syková E, Nicholson C (2008) Diffusion in brain extracellular space. Physiol Rev 88(4):1277–1340 18923183 10.1152/physrev.00027.2007 PMC2785730

[41] Di Biase MA, Zalesky A, Cetin-Karayumak S, Rathi Y, Lv J, Boerrigter D, North H, Tooney P (2021) Large-scale evidence for an association between peripheral inflammation and white matter free water in schizophrenia and healthy individuals. Schizophr Bull 47(2):542–551 33085749 10.1093/schbul/sbaa134 PMC7965083

[42] Pasternak O, Kubicki M, Shenton ME (2016) In vivo imaging of neuroinflammation in schizophrenia. Schizophr Res 173(3):200–212 26048294 10.1016/j.schres.2015.05.034 PMC4668243

[43] Ofori E, DeKosky ST, Febo M, Colon-Perez L, Chakrabarty P, Duara R, Adjouadi M, Golde TE (2019) Free-water imaging of the hippocampus is a sensitive marker of Alzheimer’s disease. Neuroimage Clin 24:101985. 10.1016/j.nicl.2019.101985 31470214 PMC6722298

[44] Centers for Disease Control and Prevention (2017) World Trade Center Health Program At A Glance. Centers for Disease Control and Prevention. https://www.cdc.gov/wtc/ataglance.html. Accessed March 22 2017 2017

[45] Dasaro CR, Holden WL, Berman KD, Crane MA, Kaplan JR, Lucchini RG, Luft BJ, Moline JM (2017) Cohort Profile: World Trade Center Health Program General Responder Cohort. Int J Epidemiol 46(2):e9. 10.1093/ije/dyv099 26094072 PMC6074831

[46] Huang C, Kritikos M, Clouston SAP, Deri Y, Serrano-Sosa M, Bangiyev L, Santiago-Michels S, Gandy S (2021) White Matter Connectivity in Incident Mild Cognitive Impairment: A Diffusion Spectrum Imaging Study of World Trade Center Responders at Midlife. J Alzheimers Dis 80:1209–1219. 10.3233/JAD-201237 33646156 PMC8150516

[47] Clouston SAP, Diminich ED, Kotov R, Pietrzak RH, Richards M, Spiro A 3rd, Deri Y, Carr M (2019) Incidence of mild cognitive impairment in World Trade Center responders: Long-term consequences of re-experiencing the events on 9/11/2001. Alzheimers Dement (Amst) 11:628–636. 10.1016/j.dadm.2019.07.006 31517025 PMC6733774

[48] Clouston SA, Kotov R, Pietrzak RH, Luft BJ, Gonzalez A, Richards M, Ruggero CJ, Spiro A 3rd (2016) Cognitive impairment among World Trade Center responders: Long-term implications of re-experiencing the 9/11 terrorist attacks. Alzheimers Dement (Amst) 4:67–75. 10.1016/j.dadm.2016.08.001 27626057 PMC5011166

[49] McKhann GM, Knopman DS, Chertkow H, Hyman BT, Jack CR, Kawas CH, Klunk WE, Koroshetz WJ (2011) The diagnosis of dementia due to Alzheimer’s disease: Recommendations from the National Institute on Aging-Alzheimer’s Association workgroups on diagnostic guidelines for Alzheimer’s disease. Alzheimers Dement 7(3):263–269 21514250 10.1016/j.jalz.2011.03.005 PMC3312024

[50] Freitas S, Simoes MR, Alves L, Santana I (2013) Montreal cognitive assessment: validation study for mild cognitive impairment and Alzheimer disease. Alzheimer Dis Assoc Disord 27(1):37–43. 10.1097/WAD.0b013e3182420bfe 22193353

[51] First MB (1997) Structured clinical interview for DSM-IV axis I disorders. Biometrics Research Department

[52] Andersson JLR, Sotiropoulos SN (2016) An integrated approach to correction for off-resonance effects and subject movement in diffusion MR imaging. Neuroimage 125:1063–1078. 10.1016/j.neuroimage.2015.10.019 26481672 PMC4692656

[53] Contributors D, Garyfallidis E, Brett M, Amirbekian BB, Rokem A, van der Walt S, Descoteaux M, Nimmo-Smith I (2014) Dipy, a library for the analysis of diffusion MRI data. Front Neuroinform 8:8 24600385 10.3389/fninf.2014.00008 PMC3931231

[54] Henriques RN, Rokem A, Garyfallidis E, St-Jean S, Peterson ET, Correia MM (2017) [Re] Optimization of a free water elimination two-compartment model for diffusion tensor imaging. BioRxiv:108795

[55] Lesh TA, Maddock RJ, Howell A, Wang H, Tanase C, Daniel Ragland J, Niendam TA, Carter CS (2021) Extracellular free water and glutathione in first-episode psychosis-a multimodal investigation of an inflammatory model for psychosis. Mol Psychiatry 26(3):761–771. 10.1038/s41380-019-0428-y 31138893 PMC6881530

[56] Nemmi F, Levardon M, Péran P (2022) Brain-age estimation accuracy is significantly increased using multishell free-water reconstruction. Hum Brain Mapp 43(7):2365–2376 35141974 10.1002/hbm.25792 PMC8996361

[57] Yeh F-C, Wedeen VJ, Tseng W-YI (2010) Generalized ${q} $-sampling imaging. IEEE Trans Med Imaging 29(9):1626–1635 20304721 10.1109/TMI.2010.2045126

[58] da Silva NM, Forsyth R, McEvoy A, Miserocchi A, de Tisi J, Vos SB, Winston GP, Duncan J (2020) Network reorganisation following anterior temporal lobe resection and relation with postsurgery seizure relapse: a longitudinal study. NeuroImage Clin 27:102320 32623138 10.1016/j.nicl.2020.102320 PMC7334605

[59] Yeh F-C, Tang P-F, Tseng W-YI (2013) Diffusion MRI connectometry automatically reveals affected fiber pathways in individuals with chronic stroke. NeuroImage Clin 2:912–921 24179842 10.1016/j.nicl.2013.06.014 PMC3777702

[60] Yeh F-C, Vettel JM, Singh A, Poczos B, Grafton ST, Erickson KI, Tseng W-YI, Verstynen TD (2016) Quantifying differences and similarities in whole-brain white matter architecture using local connectome fingerprints. PLoS Comput Biol 12(11):e1005203 27846212 10.1371/journal.pcbi.1005203 PMC5112901

[61] Yeh F-C, Badre D, Verstynen T (2016) Connectometry: a statistical approach harnessing the analytical potential of the local connectome. Neuroimage 125:162–171 26499808 10.1016/j.neuroimage.2015.10.053

[62] Yeh F-C, Tseng W-YI (2011) NTU-90: a high angular resolution brain atlas constructed by q-space diffeomorphic reconstruction. Neuroimage 58(1):91–99 21704171 10.1016/j.neuroimage.2011.06.021

[63] Jenkinson M, Bannister P, Brady M, Smith S (2002) Improved optimization for the robust and accurate linear registration and motion correction of brain images. Neuroimage 17(2):825–841 12377157 10.1016/s1053-8119(02)91132-8

[64] Clouston S, Pietrzak RH, Kotov R, Richards M, Spiro A 3rd, Scott S, Deri Y, Mukherjee S (2017) Traumatic exposures, posttraumatic stress disorder, and cognitive functioning in World Trade Center responders. Alzheimers Dement (N Y) 3(4):593–602. 10.1016/j.trci.2017.09.001 29201993 PMC5700827

[65] Benjamini Y, Hochberg Y (1995) Controlling the false discovery rate: a practical and powerful approach to multiple testing. J R Stat Soc Ser B (Methodological) 57(1):289–300

[66] Scheff SW, Price DA, Schmitt FA, Mufson EJ (2006) Hippocampal synaptic loss in early Alzheimer’s disease and mild cognitive impairment. Neurobiol Aging 27(10):1372–1384. 10.1016/j.neurobiolaging.2005.09.012 16289476

[67] Deri Y, Clouston SAP, DeLorenzo C, Gardus JD 3rd, Horton M, Tang C, Pellecchia AC, Santiago-Michels S (2021) Selective hippocampal subfield volume reductions in World Trade Center responders with cognitive impairment. Alzheimers Dement (Amst) 13(1):e12165. 10.1002/dad2.12165 33816755 PMC8011041

[68] Lioy PJ, Weisel CP, Millette JR, Eisenreich S, Vallero D, Offenberg J, Buckley B, Turpin B (2002) Characterization of the dust/smoke aerosol that settled east of the World Trade Center (WTC) in lower Manhattan after the collapse of the WTC 11 September 2001. Environ Health Perspect 110(7):703–714 12117648 10.1289/ehp.02110703 PMC1240917

[69] Yiin L-M, Millette JR, Vette A, Ilacqua V, Quan C, Gorczynski J, Kendall M, Chen LC (2004) Comparisons of the dust/smoke particulate that settled inside the surrounding buildings and outside on the streets of southern New York City after the collapse of the World Trade Center, September 11, 2001. J Air Waste Manag Assoc 54(5):515–528 15149040 10.1080/10473289.2004.10470935

[70] Galván-Arzate S, Martínez A, Medina E, Santamaría A, Ríos C (2000) Subchronic administration of sublethal doses of thallium to rats: effects on distribution and lipid peroxidation in brain regions. Toxicol Lett 116(1–2):37–43 10906420 10.1016/s0378-4274(00)00200-9

[71] Lee SH, Chen YH, Chien CC, Yan YH, Chen HC, Chuang HC, Hsieh HI, Cho KH (2021) Three month inhalation exposure to low-level PM25 induced brain toxicity in an Alzheimer’s disease mouse model. PLoS One 16(8):e0254587. 10.1371/journal.pone.0254587 34437570 PMC8389369

[72] Bromet E, Hobbs M, Clouston S, Gonzalez A, Kotov R, Luft B (2016) DSM-IV post-traumatic stress disorder among World Trade Center responders 11–13 years after the disaster of 11 September 2001 (9/11). Psychol Med 46(4):771–783. 10.1017/S0033291715002184 26603700 PMC4754831

[73] Scherrer B, Warfield SK (2010) Why multiple b-values are required for multi-tensor models. Evaluation with a constrained log-Euclidean model. In: 2010 IEEE International Symposium on Biomedical Imaging: From Nano to Macro, IEEE. 1389–1392

[74] Jordan A, Chad OP, Jean Chen J (2019) Free water mapping in diffusion MRI: How do two common approaches compare? International Society for Magnetic Resonance in Medicine Meeting

[75] Gupta CN, Turner JA, Calhoun VD (2019) Source-based morphometry: a decade of covarying structural brain patterns. Brain Struct Funct 224(9):3031–3044. 10.1007/s00429-019-01969-8 31701266

[76] Clouston SAP, Deri Y, Horton M, Tang C, Diminich E, DeLorenzo C, Kritikos M, Pellecchia AC (2020) Reduced cortical thickness in World Trade Center responders with cognitive impairment. Alzheimers Dement (Amst) 12(1):e12059. 10.1002/dad2.12059 32695871 PMC7364857

